# Genetically distinct Group B *Streptococcus* strains induce varying macrophage cytokine responses

**DOI:** 10.1371/journal.pone.0222910

**Published:** 2019-09-19

**Authors:** Rebecca A. Flaherty, Elena C. Borges, Jessica A. Sutton, David M. Aronoff, Jennifer A. Gaddy, Margaret G. Petroff, Shannon D. Manning

**Affiliations:** 1 Department of Microbiology and Molecular Genetics, Michigan State University, East Lansing, MI, United States of America; 2 Department of Microbiology and Immunology, Meharry Medical College School of Medicine, Nashville, TN, United States of America; 3 Department of Medicine, Division of Infectious Disease, Vanderbilt University Medical Center, Nashville, TN, United States of America; 4 Pathobiology and Diagnostic Investigation, Michigan State University, East Lansing, MI, United States of America; Instituto Butantan, BRAZIL

## Abstract

Group B *Streptococcus* (GBS) is an opportunistic pathogen that causes preterm birth and neonatal disease. Although GBS is known to exhibit vast diversity in virulence across strains, the mechanisms of GBS-associated pathogenesis are incompletely understood. We hypothesized that GBS strains of different genotypes would vary in their ability to elicit host inflammatory responses, and that strains associated with neonatal disease would induce different cytokine profiles than those associated with colonization. Using a multiplexed, antibody-based protein detection array, we found that production of a discrete number of inflammatory mediators by THP-1 macrophage-like cells was universally induced in response to challenge with each of five genetically distinct GBS isolates, while other responses appeared to be strain-specific. Key array responses were validated by ELISA using the initial five strains as well as ten additional strains with distinct genotypic and phenotypic characteristics. Interestingly, IL-6 was significantly elevated following infection with neonatal infection-associated sequence type (ST)-17 strains and among strains possessing capsule (cps) type III. Significant differences in production of IL1-β, IL-10 and MCP-2 were also identified across STs and cps types. These data support our hypothesis and suggest that unique host innate immune responses reflect strain-specific differences in virulence across GBS isolates. Such data might inform the development of improved diagnostic or prognostic strategies against invasive GBS infections.

## Introduction

Group B *Streptococcus* (GBS) is a common commensal of the gastrointestinal and genitourinary tracts in approximately 30% of healthy individuals [1]. Although GBS rarely causes serious infections in healthy adults, it is a leading cause of preterm birth, stillbirth, and neonatal sepsis and meningitis worldwide [1]. Maternal GBS colonization is a significant risk factor for both preterm birth and neonatal disease. Pregnancy complications can occur when GBS ascends the vaginal tract of a colonized mother, crosses the extraplacental membranes surrounding the fetus, and initiates an infection *in utero* [2]. Babies can also become infected by inhaling vaginal fluid containing GBS as they pass through the vaginal tract during delivery [3]. Neonates can develop early-onset disease (EOD), which typically presents as pneumonia and sepsis and occurs during the first week of life, or late-onset disease (LOD), which occurs from one week to three months of age [1]. To reduce the likelihood of EOD, it is recommended that colonized women receive antibiotic prophylaxis during delivery; however, prophylaxis is not effective at reducing the risk of LOD or maternal complications attributable to GBS [1].

In order to develop novel prevention and diagnostic strategies to combat infection, there is a critical need to understand how GBS modulates host immune responses. Indeed, prior studies have shown that inappropriate inflammatory signaling can contribute to adverse pregnancy outcomes such as extraplacental membrane weakening and neonatal sepsis [3,4]. The macrophage response is of particular importance for neonates, whose immature adaptive immune system forces them to rely primarily on the innate immune system to combat bacterial infections [5]. Macrophages are also present at the maternal fetal interface where they aid in the maintenance of maternal tolerance to the developing fetus and combat pathogens that have crossed the extraplacental membranes [6]. Hence, an investigation into the role that macrophages play in inflammatory signaling cascades following infection with GBS is imperative.

The GBS strain population is diverse and we and others have shown that GBS strains belonging to multilocus sequence type (ST)-17 are more common in sick neonates than colonized pregnant mothers [7–10]. Furthermore, we found that ST-17 strains and other closely related lineages were significantly more likely to cause LOD and meningitis [10]. While the mechanism(s) behind these epidemiological associations is not known, variation in strain phenotypes and host responses are likely important. For example, we have found that ST-17 strains vary in the ability to attach to and invade decidualized endometrial stromal cells, which mimic the outermost cells of placental membranes, as well as lung epithelium *in vitro* [11]. We have also shown that ST-17 strains possess unique virulence characteristics [12] and can survive longer inside macrophages than other genotypes [13], due in part to an enhanced capacity to dampen reactive oxygen species (ROS) production [14].

Because of the extensive phenotypic and genotypic variation in the GBS strain population, we sought to compare macrophage responses across 15 clinical GBS strains of varying STs and capsule types (serotypes). We hypothesized that GBS strains of different genotypes would vary in their ability to elicit host inflammatory responses, and strains associated with neonatal disease would induce different cytokine profiles than those associated with colonization. Our results show that certain inflammatory cytokines were universally induced in response to the 15 strains, while other responses were unique to specific strains, genotypes, or serotypes. The data generated through this study can be used to identify responses that represent early diagnostic indicators of maternal or neonatal complications or can be targeted for novel therapeutic intervention strategies.

## Materials and methods

### Bacterial strains

Fifteen previously characterized GBS strains were used in this study; invasive strains were from neonates with LOD or EOD [10] and colonizing strains were from women before or after childbirth [15]. Strains were selected based on ST, capsule type and source and all strains were examined for variation in phagocytic uptake in our prior study [16]. The 15 strains had the following characteristics: STs 17 (n = 4), 19 (n = 4), 12 (n = 4) and 1 (n = 3); and capsule types III (n = 8), II (n = 4), and V (n = 3). Five of these 15 strains (GB00112 (ST-17), GB00590 (ST-19), and GB00653 (ST-12)) from colonized mothers) and two (GB00411 (ST-17) and GB00037 (ST-1) from neonates with EOD) were used for the antibody array analyses, while the remaining 10 strains were evaluated using ELISAs for key cytokines identified in the arrays. Strains were grown in Todd-Hewitt broth (THB) at 37°C for 16–20 hours, sub-cultured to THB and grown to log phase (OD_600_ of 0.4), washed in sterile PBS, and resuspended in RPMI 1640 (Gibco) prior to infection.

### THP-1 cell culture and infection

THP-1 monocyte-like cells (ATCC TIB-202) were cultured in RPMI 1640 medium supplemented with 2mM L-glutamine (Gibco), 10% fetal bovine serum (FBS; Atlanta Biologicals), and 1% penicillin/streptomycin (Gibco) at 37ºC with 5% carbon dioxide. Cells were differentiated into macrophages by incubation with 100nM phorbol 12-myristate 13-acetate (PMA; Sigma) in RPMI medium with 2% FBS for 24hr as previously described [13]. The cells were seeded at a density of 1x10^6^ cells per well in 24 well plates or at 4x10^6^ cells per well in 6 well plates.

Prior to bacterial infection, PMA-treated THP-1 cells were washed with PBS and fresh RPMI 1640 media without supplements was added. Log phase GBS cultures were centrifuged, washed in PBS, and resuspended in RPMI 1640. Optical densities (OD_600 nm_) were normalized based on experimentally determined CFU to OD_600_ corollaries for each strain to achieve the desired multiplicity of infection (MOI). All strains used in this study had identical growth rates in the experimental conditions utilized; no statistically significant differences were observed. THP-1 cells were infected with the normalized cultures of GBS at a MOI of 10 bacteria per host cell, which equated to 1x10^7^ CFU/well in 24 well plates or 4x10^7^ CFU/well in 6 well plates. The infected cells were incubated at 37°C with 5% CO_2_ for 1hr. Extracellular bacteria and media were aspirated, and the cells were washed with PBS and RPMI 1640 supplemented with 2% FBS, 100μg/ml gentamicin (Gibco), 5μg/ml penicillin G (Sigma). The cells were incubated in 100nM PMA at 37°C with 5% CO_2_ for 24hrs prior to collecting the culture media for cytokine profiling. This time point was selected to allow for the detection of both rapid and delayed cytokine responses.

### Cytokine arrays and cytokine detection by ELISA

Supernatant collection, processing, and analysis methods were adapted from those described previously by Flaherty et al. [17]; modifications included the time point of sample collection, the human cell type analyzed and the bacterial species tested. To select the experimental conditions for the arrays, IL-1β was selected as a representative cytokine, and its levels were assessed by ELISA with four GBS strains at 1, 3, 5, 18 and 24hrs post-antibiotic treatment to determine the time when the most robust responses could be seen (data not shown). The greatest cytokine levels were observed after 24hrs and therefore, this time point was chosen for the subsequent assays.

Culture media was collected 24hrs after antibiotics were added and centrifuged (2400 rcf for 10 mins) to remove bacteria and cellular debris; supernatants were stored at -20˚C. Samples were thawed on ice and centrifuged at 16,000 rcf for 5 mins to remove any remaining debris. The Abcam Human Cytokine Antibody Array Kit (ab133998), which allows for the detection 80 human cytokines, was performed in duplicate with two independent biological replicates per condition and used according to the manufacturer’s instructions to determine relative cytokine levels per sample with an Amersham Imager 600 (GE Life Sciences) (S1 Fig). Cytokine functions were defined by UniProt (http://www.uniprot.org/) (S1 Table). Densitometry was performed using ImageJ to determine relative protein levels of each cytokine; densitometry values were corrected using internal positive controls for each array, and the normalized values were graphed (S2 Fig). Densitometry values from each infection condition were compared to the corresponding mock infection condition to calculate fold changes. Data from the two independent biological replicates were then averaged for each cytokine (S3 Fig). Significance compared to mock infection was assessed by ANOVA and post-hoc Dunnett’s testing using normalized densitometry values for all conditions as well as fold change values compared to mock infection for each infection condition (the mock infection was set to 1.0 for fold change comparisons). Additionally, fold changes between the two array sets were compared, and cytokines for which a fold change of ≥1.5 was observed in both independent array replicates for that infection condition are indicated in bold.

ELISAs were used to confirm a subset of cytokines identified in the antibody arrays using supernatants from additional independent biological replicates for 15 GBS strains. Comparisons were made relative to mock infection. To ensure that inclusion of PMA had no impact on cell signaling responses, we compared responses from two mock infections; one included the addition of PMA throughout the experiment and another included the addition of PMA exclusively during the initial differentiation step. No significant differences in cytokine levels for the six cytokines were observed among all 15 strains between the cells treated once or continuously with PMA (data not shown). Only the PMA-treated results are shown.

The following ELISA kits were used according to the manufacturer’s instructions: IL-1β Human ELISA kit: EH2IL1B2 (Thermo-Fisher Scientific), MIG Human ELISA kit: EHCXCL9 (Thermo-Fisher Scientific), IL-6 Human ELISA kit: KHC0061 (Thermo-Fisher Scientific), MCP-2 Human ELISA kit: EHCCL8 (Thermo-Fisher Scientific), RANTES Human ELISA kit: EHRNTS (Thermo-Fisher Scientific), and IL-10 Human ELISA kit: ab100549 (Abcam). MIG is also referred to as CXCL9 (C-X-C motif chemokine ligand, while MCP-2 and RANTES are referred to as CCL8 (C-C motif chemokine 8) and CCL5 (C-C motif chemokine ligand 5), respectively (https://www.genenames.org/). Data from 3 to 5 independent biological replicates were pooled to quantify the average cytokine concentrations (pg/mL) per condition for each of the 15 strains and to calculate the standard deviation of the mean. Significance was determined in each assay by ANOVA and post-hoc Dunnett’s tests relative to mock infection.

Additionally, cytokine levels from strains of the same ST, capsule type, and source (colonizing or invasive) were pooled to identify significant differences by strain characteristic using ANOVA followed by post-hoc Tukey’s tests. Samples for the additional 10 strains not included in the cytokine array were collected and analyzed separately from the five strains included in the array. Because these additional samples were not part of the same set of biological replicates, a normalization step was performed to account for experimental variability. The average cytokine signal levels for each cytokine tested by ELISA were determined for the two sets of strains (initial 5 vs. additional 10). These average signal values were compared to calculate a ratio (e.g., IL-6 average for set 1 = 40.9 pg/mL; average for set 2 = 45.08 pg/mL; ratio of the two sets = 0.9), and the values for the second set of strains were adjusted based on the difference in the average signal value between the two sets (e.g., set 2 values multiplied by 0.9). This adjustment allowed for a more accurate representation of the two data sets together on the same graph for each cytokine.

## Results

### GBS induces distinct inflammatory cytokine signaling responses in macrophages

**Fig 1:**
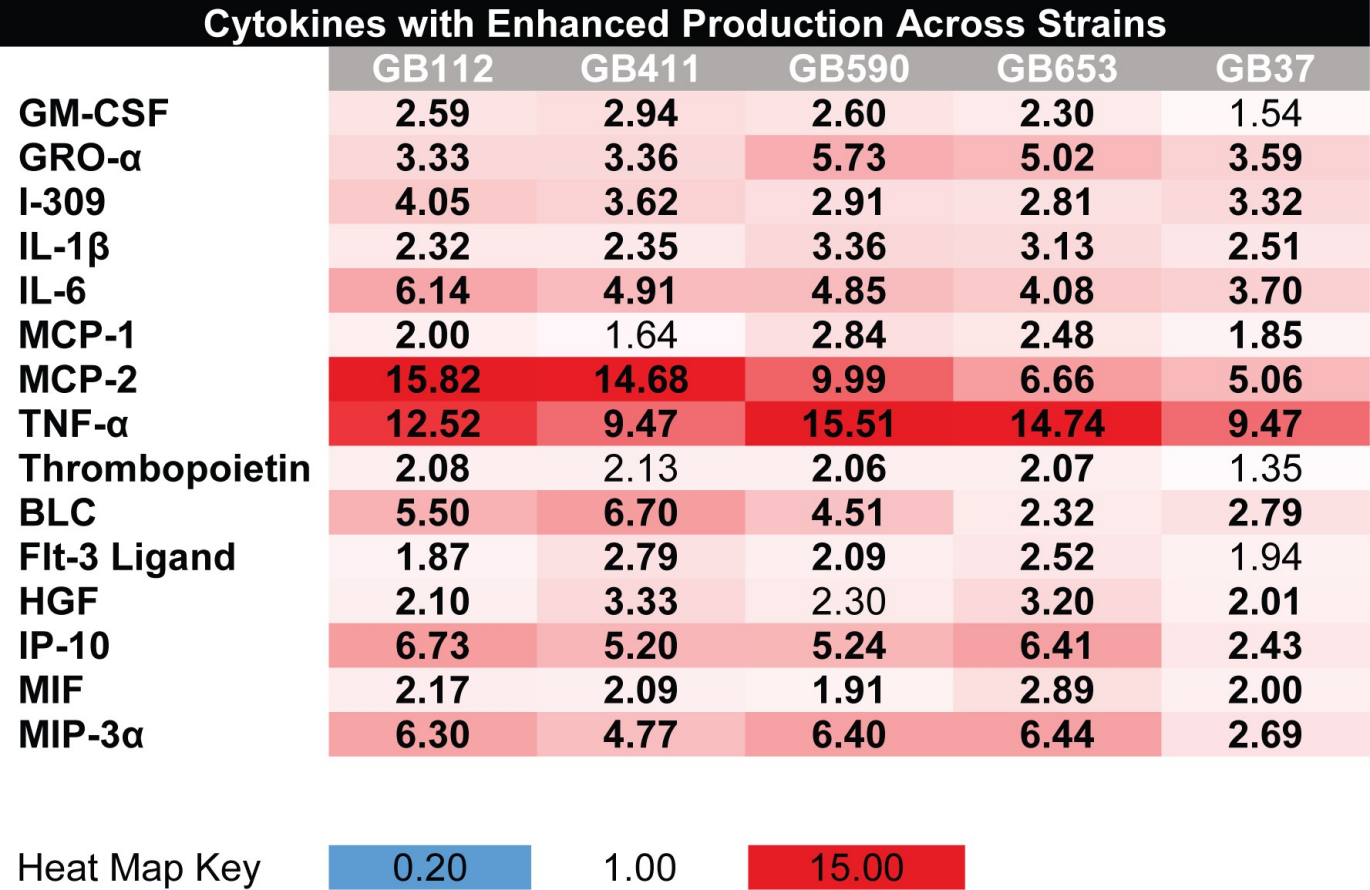
Macrophage cytokine responses induced by all five GBS strains. Cytokines with increased production relative to mock infection in both cytokine array replicates during infection with GBS strains are shown in red, and cytokines with decreased production are shown in blue. Values represent the average fold change relative to mock infection from the two independent array replicates for each condition. Bolded values represent cytokines that had fold changes of ≥1.5 above or below the mock infection in both array replicates.

Array data for the five GBS strains found that a subset of inflammatory cytokines were induced in most strains in response to infection. Cytokines GM-CSF, GRO-α, I-309, IL-1β, IL-6, MCP-1, MCP-2, TNF-α, thrombopoietin, BLC, Flt-3 ligand, HGF, IP-10, MIF, and MIP-3α were elevated by ≥1.5 fold relative to mock infection in both independent biological replicates of the cytokine array (Fig 1).

### Distinct GBS strain types elicit varying macrophage cytokine responses

**Fig 2:**
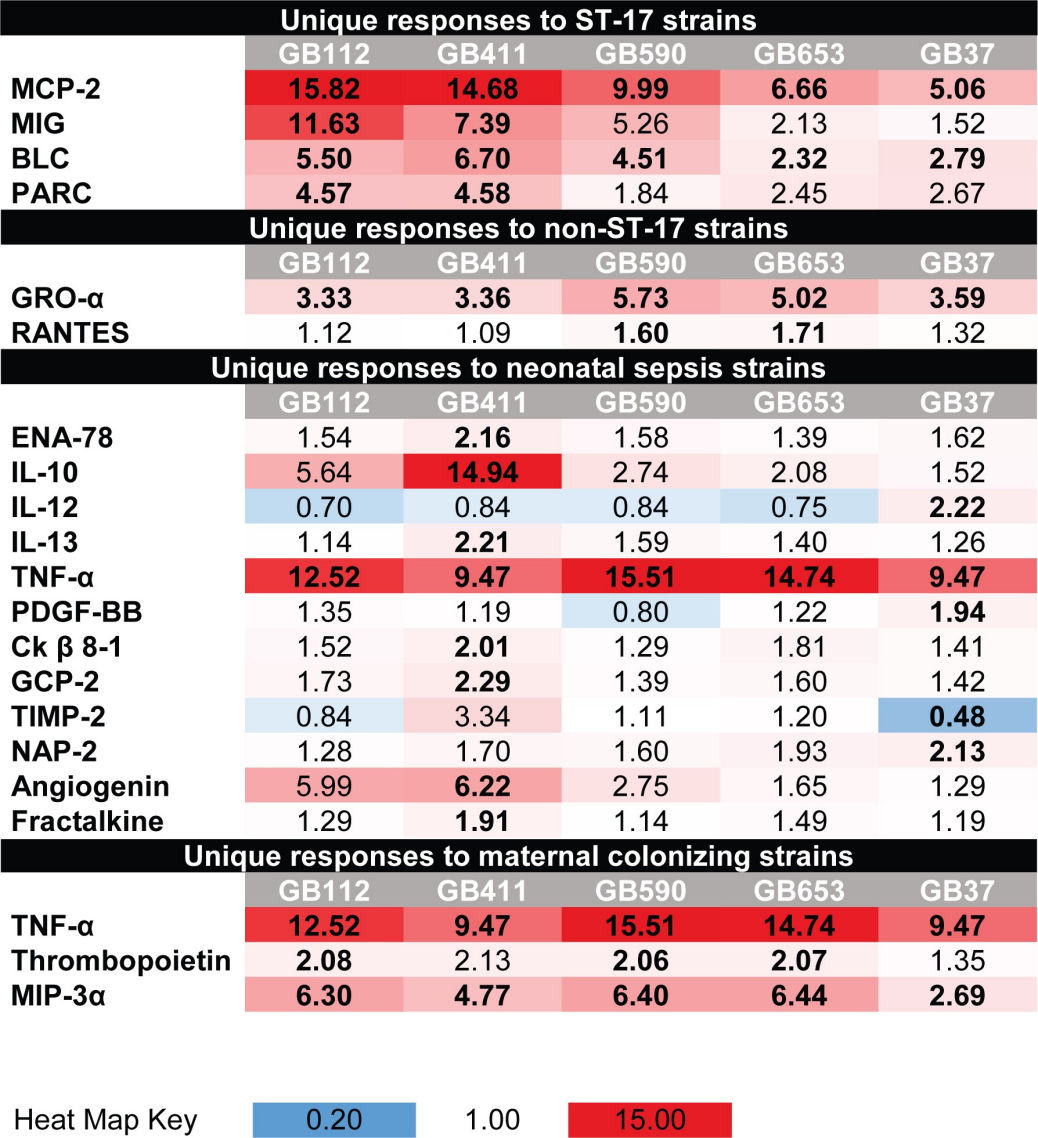
Macrophage cytokine responses to specific GBS strains or genotypes. Cytokines with increased production relative to mock infection in both cytokine array replicates during infection with GBS strains are shown in red, and cytokines with decreased production are shown in blue. Values represent the average fold change relative to mock infection from the two independent array replicates for each condition. Bolded values represent cytokines that had fold changes of ≥1.5 above or below the mock infection in both array replicates. (Top) Cytokines that were elevated in response to ST-17 strains (GB112 and GB411). (Middle) Cytokines that were elevated in response to non-ST-17 strains (GB590, GB653, and GB37). (Bottom) Cytokines that differed for one or both neonatal sepsis isolates (GB411 and GB37).

In addition to these shared macrophage responses, some changes in cytokine production were unique to strains of specific genotypes or were induced only by strains isolated from infected neonates or from colonized mothers. MCP-2, MIG, BLC, and PARC were more highly elevated in the two ST-17 strains relative to the other STs, while GRO-α and RANTES were more highly elevated in the non-ST-17 strains (Fig 2). Cytokines that had unique and consistent changes in production in response to one or both of the invasive neonatal strains included ENA-78, IL-10, IL-12, IL-13, TNF-α, angiogenin, PDGF-BB, Ck β 8–1, Fractalkine, GCP-2, IGFBP-1, PLFG, TGF-β3, and TIMP-2 (Fig 2). These unique responses for both ST-17 strains and for sepsis isolates may contribute to the ability of these strains to deregulate the host immune response and induce particularly severe disease outcomes compared to other GBS strains.

### Confirmation of cytokine array results by ELISA

**Fig 3:**
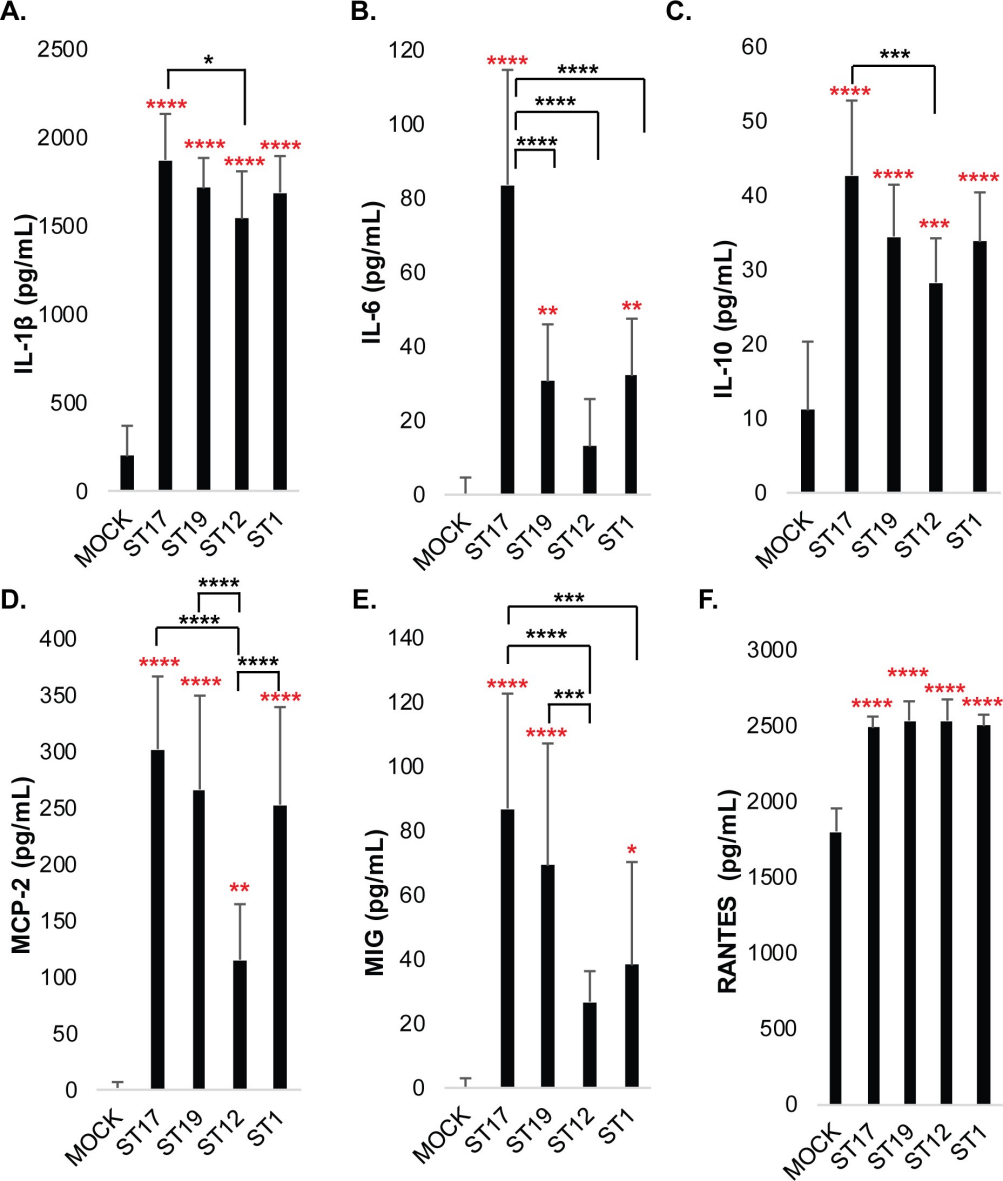
ELISA results demonstrate sequence type-specific modulation of macrophage inflammatory signaling during GBS infection. THP-1 cells were infected with GBS at MOI of 10 for 1hr. Extracellular bacteria were then removed, antibiotics were applied, and cytokines were collected 24hrs later for analysis. Data from three to five independent biological replicates were pooled for each of 15 strains to determine the average cytokine concentrations (pg/mL) produced following each of the infection conditions. Concentration values for strains of the same sequence type were then pooled to assess differences between the four sequence types analyzed. The average and standard deviation of each sequence type were plotted for comparison, and significance was determined by ANOVA, followed by post-ANOVA Tukey’s tests to compare the mean of each group to the mean of each of the other groups as indicated. All cytokines tested had ANOVA p-values <0.0001. Significant differences compared to mock infection are indicated with red asterisks, and significant differences compared to other strain groups are indicated with black asterisks.

**Fig 4:**
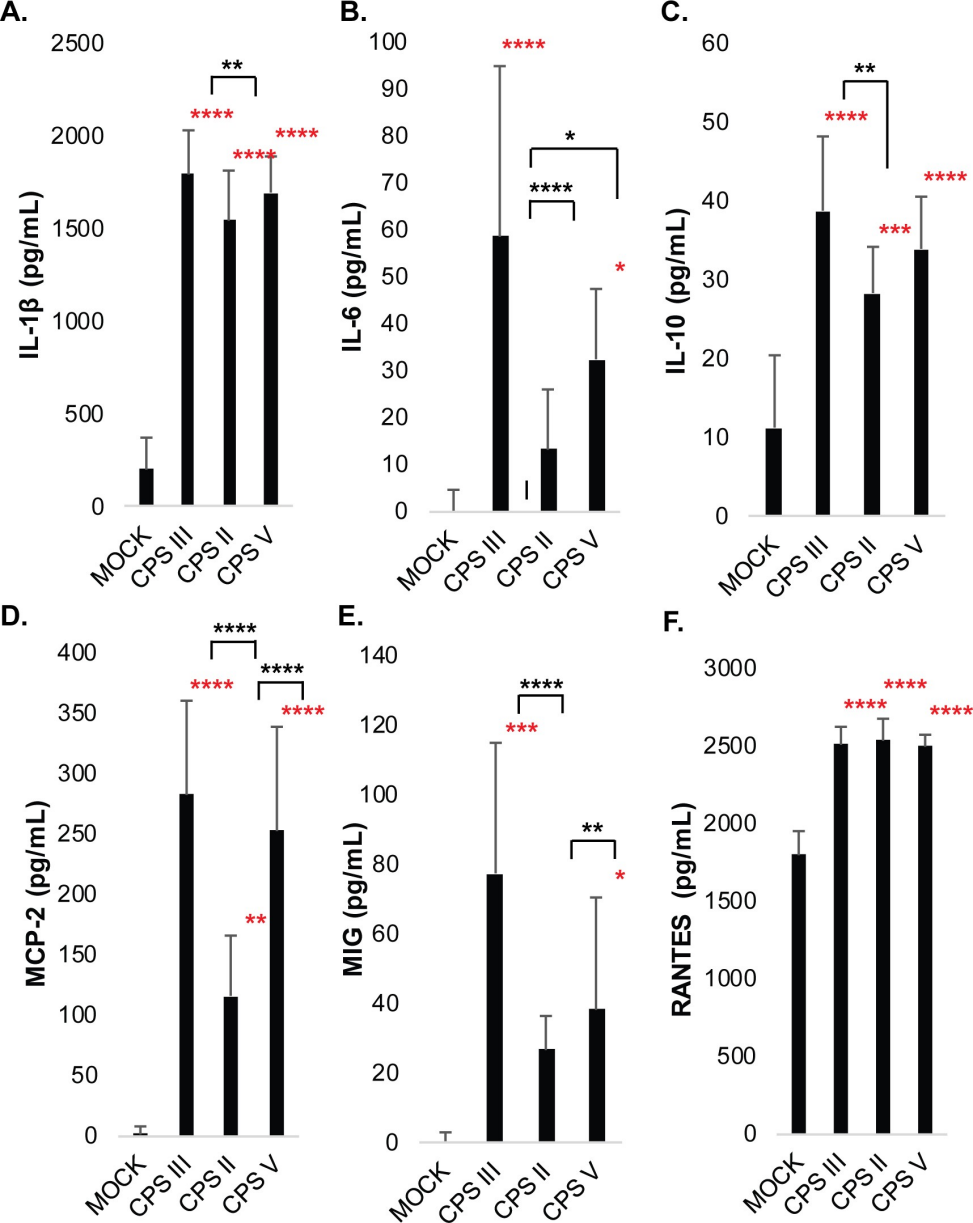
ELISA results demonstrate capsule type-specific modulation of macrophage inflammatory signaling during GBS infection. THP-1 cells were infected with GBS at MOI of 10 for 1hr. Extracellular bacteria were then removed, antibiotics were applied, and cytokines were collected 24hrs later for analysis. Data from three to five independent biological replicates were pooled for each of 15 strains to determine the average cytokine concentrations (pg/mL) produced following each of the infection conditions. Concentration values for strains of the same capsule type were then pooled to assess differences between the three capsule types analyzed. The average and standard deviation of each capsule type were plotted for comparison, and significance was determined by ANOVA, followed by post-ANOVA Tukey’s tests to compare the mean of each group to the mean of each of the other groups as indicated. All cytokines tested had ANOVA p-values <0.0001. Significant differences compared to mock infection are indicated with red asterisks, and significant differences compared to other strain groups are indicated with black asterisks.

Six representative cytokines were selected for confirmation by ELISA based on the cytokine array results. The cytokine arrays had indicated high protein levels of IL-1β in response to the first five GBS strains tested, with the highest levels being induced by GB00590 and GB00653 (S3 Fig). The IL-1β ELISA confirmed robust and significant IL-1β production compared to mock infection for all 15 GBS strains (S4A Fig). When results from strains of the same ST or capsule (CPS) type were pooled and averaged, ST-17 strains induced significantly higher IL-1β than ST-12 strains (Fig 3A) and CPS III strains induced significantly higher IL-1β than CPS II strains (Fig 4A). There was no significant difference between strains isolated from colonized mothers compared with those isolated from infected neonates.

As predicted by the arrays, increased levels of IL-6 were also observed in response to all 15 strains, with the highest cytokine levels being induced by the four ST-17 strains (GB00112, GB00411, GB00097, and GB00418), and moderate levels being induced by the other 11 strains (S4B Fig). Stratifying by ST demonstrated that ST-17 strains induced significantly higher IL-6 levels than strains belonging to all other STs (Fig 3B) as did CPS III strains relative to CPS II and CPS V strains (Fig 4B). No significant difference in IL-6 induction was observed between strains isolated from colonized mothers versus infected neonates.

The cytokine array results also showed an increased level of IL-10 across conditions compared to mock infection, with the greatest level being induced by ST-17 strains (S3 Fig). The ELISA data also indicated significantly enhanced IL-10 production in response to all 15 strains, though the highest levels were observed in response to the ST-17 strains (S4C Fig). Indeed, averaging results from strains of the same ST indicated that ST-17 strains induced significantly higher IL-10 production than ST-12 strains. Similarly, CPS III strains, which represent ST-17 and ST-19 strains, induced significantly higher IL-10 levels than CPS II strains (Figs 3C and 4C). IL-10 production was not significantly different between maternal colonizing and invasive neonatal strains.

Substantially increased levels of MCP-2 was also observed in response to most GBS strains, which was also confirmed by ELISA. The highest levels of MCP-2 were observed in response to challenge with the ST-17 strains (Fig 3D). ST-19 and ST-1 strains, however, also induced high levels of MCP-2 on average, and all three STs induced significantly more MCP-2 than ST-12 strains. A similar difference was observed after stratifying by capsule type; significantly greater MCP-2 induction was seen in CPS III and CPS V strains compared to CPS II strains (S4D Fig). There was no significant difference in MCP-2 induction between colonizing and invasive strains.

The cytokine arrays also predicted enhanced levels of MIG in response to ST-17 infection, which was confirmed by ELISA (Fig 3E). All four ST-19 strains induced significantly enhanced levels of MIG compared to mock infection as well, though only one ST-12 and one ST-1 strain induced significantly higher levels of MIG (S4E Fig). Averaged cytokine levels from the pooled STs indicated that ST-17 strains induced significantly more MIG than ST-12 or ST-1 strains, and that ST-19 strains also induced significantly greater MIG levels than ST-12 strains (Fig 3E). Averaged CPS III and CPS V strains were found to induce significantly greater MIG levels than CPS II strains (Fig 4E). There was no significant difference in MIG induction between strains isolated from colonized mothers compared with those isolated from infected neonates (not shown). RANTES was induced at high levels in response to both infection and mock infection conditions, with the GB00590 and GB00653 strains inducing the highest levels (S3 Fig). These trends were confirmed by ELISA (S4F Fig), with all 15 strains producing significantly higher levels of RANTES than the mock infection, and with the GB00590 and GB00653 strains inducing the most robust cytokine levels. We did not observe any significant differences in RANTES levels between averaged STs or CPS types (Figs 3F and 4F), though RANTES was significantly higher in the invasive strains when compared to the averaged colonizing strain responses (S5 Fig).

## Discussion

Through the use of an array specific for 80 cytokines, we found that five genetically diverse clinical isolates of GBS induced a range of pro- and anti-inflammatory cytokine responses following infection of THP-1 cells. A subset of the responses identified in the array were strikingly similar and likely represent general or shared cytokine responses that are induced in macrophages by other GBS strains as well (Fig 1). Importantly, validation by ELISA supports this conclusion and indicates that strains of the same ST and/or serotype do, in fact, tend to induce similar cytokine responses (Figs 3 and 4 and S4 Fig). One of the most significantly upregulated cytokines following infection with all five strains was TNF-α, a potent pyrogen that can induce cell death and has been linked to tissue damage and sepsis previously [18]. Additional cytokines such as GRO-α, I-309, IL-1β, IL-6, MCP-2, IP-10, and MIP-3α, which play critical roles in immune cell activation and recruitment, were also upregulated in response to GBS infection and may be important for activating downstream immune responses (S6 Fig). Although these responses displayed similar trends for all five of the initial strains tested using the array, differences in the magnitude of the responses were observed and could be due to phenotypic and/or genotypic differences in the GBS strains examined. These results are consistent with related studies in murine models and adult or cord blood monocytes demonstrating enhanced TNFα, IL-6, and IL-1β in response to GBS infection [19–22].

Some cytokines, particularly those involved in multifunctional pro-inflammatory responses, negative regulation of the inflammatory response or in the recruitment of specific immune cell types, such as B cells and T cells, were strongly induced in response to specific strains (S6 Fig), which is consistent with prior studies. One report, for example, compared induction of the IL-6 pro-inflammatory cytokine by cord blood monocytes following exposure to heat-killed GBS strains isolated from babies with sepsis or those who were asymptomatically colonized; enhanced IL-6 production was observed in response to sepsis isolates [23]. Likewise, we observed the greatest increases in IL-6 production by THP-1 cells infected with ST-17 strains, a genotype that has been linked to neonatal infection [7–10]. CPS III strains also had high levels of IL-6 induction, which is notable given that prior studies have observed a relationship between serotype III and invasive disease in neonates and infants [24]. Levels of IL-10, an anti-inflammatory cytokine, were also most highly elevated in response to the ST-17 and CPS III strains examined. These data support the findings of a recent meta-analysis that found lower IL-6 levels to be associated with a better sepsis prognosis and higher IL-10 levels to be a predictor of more severe disease [18].

Another study has reported elevated levels of IL-1β, IP-10, I-309, and MCP-2 in blood samples from newborns with sepsis induced by a variety of bacterial pathogens compared to healthy babies [25]; all of these cytokines were enhanced in the array analysis (Figs 1 and 2). Although nearly all the strains induced significant increases in MCP-2 and IL-1β compared to mock infection using ELISAs, these cytokines were more robustly induced by the four ST-17 strains. Indeed, increased expression of IL-1β as well as TNFα and IL-6 has been described following infection from peritoneal exudate cells and spleen cells in mice 24 hrs post-infection [19]. The cytokine array analysis also identified cytokines MIG, BLC and PARC, which enhance B and T cell activation and recruitment, to be induced at higher levels in response to the ST-17 strains; ELISAs confirmed MIG levels to be significantly higher in the four ST-17 strains relative to strains of ST-12 and ST-1. Hence, quantification of sepsis-associated cytokines such as TNF-α, IL-6, IL-10, and MCP-2, as well as cytokine activators of the adaptive immune response, which can be induced in response to more virulent GBS strains, may represent useful indictors of GBS-associated infections and help identify high-risk disease cases.

Some of the most unique responses observed by cytokine array occurred in response to the two strains isolated from infants with sepsis (GB00411 and GB00037). Both strains induced responses that could dampen or deregulate inflammatory signaling through induction of the anti-inflammatory cytokine IL-10, and, in the case of the GB00411 strain, induction of the anti-inflammatory cytokine IL-13. This strain also caused production of angiogenin, which regulates tissue vascularization, and induced production of immune cell activators such as ENA-78, Fractalkine and GCP-2. Cytokines that regulate embryonic and placental development, such as PLGF and TGF-β3 (S5 Fig) were induced as well. Although both invasive isolates induced high levels of TNF-α production, the fold change from mock infection was three to six-fold lower than what was observed for the other three strains recovered from colonized mothers. This difference could be due to the enhanced production of anti-inflammatory cytokines in response to these strains and may indicate that GBS can prevent or delay an effective immune response. Overall, the invasive strain GB00037, exhibited a similar cytokine profile to the invasive strain GB00411, though the levels for most cytokines tended to be lower. Consequently, the GB00037 invasive strain may not trigger as robust of a response, which could be due to the lack of hemolytic pigmentation described [26] or other factors unique to this strain.

The notable differences observed between strains coupled with epidemiological data suggest that invasive strains or STs and serotypes associated with severe disease have specific characteristics that can trigger more potent or potentially harmful immune responses. It is likely that these responses vary depending on the timing and duration of infection as well as the primary infection site. Some strains may contribute to more rapid responses, perhaps resulting in miscarriage or EOD, while others take longer to initiate, and may result in complications associated with LOD. In this study, we only examined cytokine responses after 24 hrs post-infection since our aim was to detect both early and delayed responses. We anticipate, however, that other differences in cytokine production might also be observed between strains at earlier time points, which could be indicative of more rapid induction of responses or inhibition. Early inhibition of specific cytokines has been linked to delayed or inappropriate immune responses for several infections [27], which could also be important for GBS. Additional studies are greatly needed to better understand the timing and duration of specific responses *in vitro* and *in vivo* using different animal model systems. Even though we have identified important differences in cytokine responses across strains, additional strains from different sources or representing other ST/CPS combinations should be examined to classify the range of GBS responses and identify those that are most important for deregulating host immunity, inflammation and neonatal and maternal infections.

Because it has been shown that faulty inflammatory signaling may lead to extraplacental membrane weakening and premature membrane rupture [28], we expect that some pro-inflammatory signaling responses identified in this study may contribute to those outcomes. Several cytokines such as Flt-3 ligand, PLGF, and TGF-β3, which were induced following infection by at least one of the two invasive strains (S6 Fig), have been classified as regulators of embryonic and placental development. Many of the GBS strains also contributed to significant changes in the production of cytokines that regulate tissue vascularization, platelet production, cellular growth, differentiation and survival, hematopoietic precursor development, and metalloproteinase activity (S5 Fig). Infection-induced deregulation of these key processes could severely impact fetal development, and altered production of some of these cytokines has been linked to sepsis previously [18]. GBS also caused changes in cytokines that regulate key functions in cells of the central or peripheral nervous systems, such as NT-3, which could play a role in meningitis or other neurological complications associated with GBS infection (S6 Fig).

Although we did not confirm each cytokine by ELISA, the set of six cytokines evaluated showed highly similar trends to those observed in the arrays and provided useful targets for examining these key responses in other GBS strains. We anticipate that most array hits with consistent trends across the two array replicates are likely to represent true positives. However, cytokines that were markedly altered compared to mock infection in only one of the two arrays may still have a role in the macrophage response to GBS infection, but additional verification and study is required.

Collectively, these data suggest that more virulent strains, STs, or serotypes of GBS elicit different cytokine responses compared to less virulent strains. Some of the differences might be partly due to differences in GBS uptake by or survival within macrophages, or in the differential expression of key virulence factors, which we have demonstrated previously [13,16]. Future work will involve the identification of strain-specific virulence factors that impact the differential induction of cytokines by macrophages *in vitro* and *in vivo* using a murine model of GBS infection. Together, these studies will enhance understanding of how invasive and colonizing strains of GBS differ in their ability to induce host responses that can initiate or exacerbate the disease process. Such discoveries will aid in the identification of new biomarkers and vaccine targets that can guide prevention practices and therapeutic intervention strategies aimed at reducing diseases caused by GBS.

## Supporting information

S1 Fig Cytokine arrays of THP-1 cells infected with five GBS strains. Differentiated THP-1 cells were infected with GBS strains for 1hr at a MOI of 10 bacteria per host cell. Cytokine levels in the collected cell culture supernatants were determined by cytokine array. Blots from one representative exposure are shown for each condition for each array set along with a key identifying each spot in the array.
(PDF)

S2 Fig Graph of densitometry values from THP-1 cytokine arrays following infection with five GBS strains. Differentiated THP-1 cells were infected with GBS strains for 1hr at a MOI of 10 bacteria per host cell. Densitometry values for each cytokine are shown from each of the two array replicates. The internal positive control values were averaged for individual arrays and used to normalize each array condition to allow accurate comparisons between mock and infection conditions. Negative control values from each array are also shown to indicate the lower limit of detection for the array.
(PDF)

S3 Fig Fold changes in cytokine production in THP-1 cells in response to GBS infection. Average fold changes for all cytokines included in the cytokine arrays are shown, along with ANOVA p-values based on normalized densitometry values from the two array replicates and ANOVA p-values based on fold changes (for fold change comparisons the mock infection values were set to 1.0). Cytokines with increased production relative to mock infection in both cytokine array replicates during infection with GBS strains are shown in red, and cytokines with decreased production are shown in blue. Values represent the average fold change relative to mock infection from the two independent array replicates for each condition. Bolded values represent cytokines that had fold changes of ≥1.5 above or below the mock infection in both array replicates.
(PDF)

S4 Fig ELISA results confirming strain-specific modulation of macrophage inflammatory signaling during GBS infection. THP-1 cells were infected with GBS at MOI of 10 for 1hr. Data from three to five independent biological replicates were pooled to determine the average cytokine concentrations (pg/mL) produced following each of the infection conditions shown. The average and standard deviation of each condition were plotted for comparison, and significance was determined by ANOVA, followed by post-ANOVA Dunnett’s tests to compare the mean of each condition to the mean of the mock infection condition. All cytokines tested had ANOVA p-values <0.0001.
(PDF)

S5 Fig ELISA results confirming RANTES signaling among invasive and colonizing GBS strains. THP-1 cells were infected with GBS at MOI of 10 for 1hr. Data from three to five independent biological replicates were pooled to calculate the average RANTES concentration (pg/mL) produced following infection with invasive or colonizing GBS strains. The standard deviation was plotted for comparison, and significance was determined by ANOVA using Tukey’s test of multiple comparisons. p-values <0.0001 are indicated.
(JPG)

S6 Fig Fold changes in cytokine production grouped by cytokine function. Cytokines with increased production relative to mock infection in both cytokine array replicates during infection with GBS strains are shown in red, and cytokines with decreased production are shown in blue. Values represent the average fold change relative to mock infection from the two independent array replicates for each condition. Bolded values represent cytokines that had fold changes of ≥1.5 above or below the mock infection in both array replicates. Major cytokine functions were classified via the UniProt Database (http://www.uniprot.org/).
(PDF)

S1 Table Functional annotations of cytokines examined in the study. Major functions were defined by and extracted from the UniProt Database (http://www.uniprot.org/). **Abbreviations:** NKs–Natural Killer Cells; DCs–Dendritic Cells; MMP–Matrix Metalloproteinases; TIMPs–Tissue inhibitors of metalloproteinases; SC–Stem Cells.
(PDF)
