# Supplementary material for: Genetically distinct Group B Streptococcus strains induce varying macrophage cytokine responses
Source: PLoS One. 2019 Sep 19;14(9):e0222910. doi: 10.1371/journal.pone.0222910 (PMC6752832; doi:10.1371/journal.pone.0222910)
Supplement: S1 Fig — Differentiated THP-1 cells were infected with GBS strains for 1hr at a MOI of 10 bacteria per host cell. Cytokine levels in the collected cell culture supernatants were determined by cytokine array. Blots from one representative exposure are shown for each condition for each array set along with a key identifying each spot in the array. (PDF) [file pone.0222910.s001.pdf]

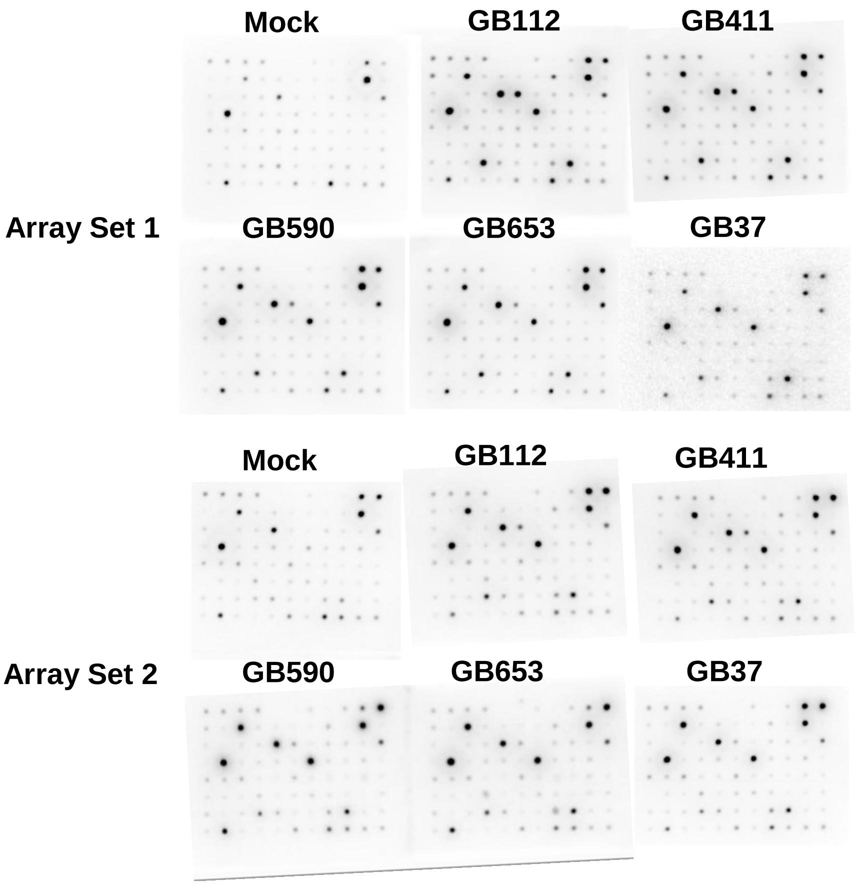

Cytokine Array Key

POS	POS	POS	POS	NEG	NEG	ENA-78	GCSF	GM-CSF	GRO	GRO-α
I-309	IL-1α	IL-1β	IL-2	IL-3	IL-4	IL-5	IL-6	IL-7	IL-8	IL-10
IL-12	IL-13	IL-15	IFN-γ	MCP-1	MCP-2	MCP-3	MCSF	MDC	MIG	MIP-1b
MIP-1δ	RANTES	SCF	SDF-1	TARC	TGF-β1	TNF-α	TNF-β	EGF	IGF-I	Angiogenin
Oncostatin M	Thrombo-poietin	VEGF	PDGF-BB	Leptin	BNDF	BLC	Ck β 8-1	Eotaxin	Eotaxin-2	Eotaxin-3
FGF-4	FGF-6	FGF-7	FGF-9	Flt-3 Ligand	Fractal-kine	GCP-2	GDNF	HGF	IGFBP-1	IGFBP-2
IGFBP-3	IGFBP-4	IL-16	IP-10	LIF	LIGHT	MCP-4	MIF	MIP-3α	NAP-2	NT-3
NT-4	Osteopontin	Osteo-protegerin	PARC	PIGF	TGF-β2	TGF-β3	TIMP-1	TIMP-2	POS	POS
