# Supplementary material for: Genetically distinct Group B Streptococcus strains induce varying macrophage cytokine responses
Source: PLoS One. 2019 Sep 19;14(9):e0222910. doi: 10.1371/journal.pone.0222910 (PMC6752832; doi:10.1371/journal.pone.0222910)
Supplement: S3 Fig — Average fold changes for all cytokines included in the cytokine arrays are shown, along with ANOVA p-values based on normalized densitometry values from the two array replicates and ANOVA p-values based on fold changes (for fold change comparisons the mock infection values were set to 1.0). Cytokines with increased production relative to mock infection in both cytokine array replicates during infection with GBS strains are shown in red, and cytokines with decreased production are shown in blue. Values represent the average fold change relative to mock infection from the two independent array replicates for each condition. Bolded values represent cytokines that had fold changes of ≥1.5 above or below the mock infection in both array replicates. (PDF) [file pone.0222910.s003.pdf]

	GB112	GB411	GB590	GB653	GB37	p-value densitometry	p-value fold change
**ENA-78**	1.54	**2.16**	1.58	1.39	1.62	0.2791	0.6690
**GCSF**	1.28	1.39	1.33	2.28	1.57	0.6638	0.9878
**GM-CSF**	**2.59**	**2.94**	**2.60**	**2.30**	1.54	0.6637	0.2893
**GRO**	**1.95**	1.59	1.88	1.84	**1.60**	0.6988	0.1490
**GRO-α**	**3.33**	**3.36**	**5.73**	**5.02**	**3.59**	0.4739	0.6191
**I-309**	**4.05**	**3.62**	**2.91**	**2.81**	**3.32**	0.1549	0.4160
**IL-1α**	1.02	1.10	0.88	1.18	1.19	0.9255	0.9559
**IL-1β**	**2.32**	**2.35**	**3.36**	**3.13**	**2.51**	0.6709	0.0058
**IL-2**	0.79	0.62	1.12	1.19	0.84	0.8707	0.8916
**IL-3**	1.70	1.60	**2.00**	**1.68**	1.44	0.7869	0.7209
**IL-4**	1.35	1.47	1.95	1.96	2.10	0.9028	0.9414
**IL-5**	1.10	1.03	1.11	1.50	1.22	0.4048	0.8974
**IL-6**	**6.14**	**4.91**	**4.85**	**4.08**	**3.70**	0.0853	0.4429
**IL-7**	0.63	0.82	0.63	0.64	0.80	0.9470	0.8843
**IL-8**	1.11	1.12	1.51	1.29	1.18	0.1707	0.5844
**IL-10**	5.64	14.94	2.74	2.08	1.52	0.2895	0.5661
**IL-12**	0.70	0.84	0.84	0.75	**2.22**	0.7390	0.1323
**IL-13**	1.14	**2.21**	1.59	1.40	1.26	0.6608	0.4471
**IL-15**	0.99	0.95	1.28	1.39	1.50	0.9903	0.9562
**IFN-γ**	1.02	1.48	0.99	1.17	1.04	0.5666	0.9011
**MCP-1**	**2.00**	1.64	**2.84**	**2.48**	**1.85**	0.0057	0.1213
**MCP-2**	**15.82**	**14.68**	**9.99**	**6.66**	**5.06**	0.0311	0.0843
**MCP-3**	1.40	1.02	1.32	1.93	1.99	0.6534	0.7532
**MCSF**	1.55	1.11	1.69	1.10	1.35	0.8162	0.9352
**MDC**	1.25	1.22	1.94	1.87	1.86	0.3960	0.7228
**MIG**	11.63	7.39	5.26	2.13	1.52	0.2738	0.5507
**MIP-1b**	1.19	1.04	1.90	1.91	1.63	0.0189	0.3756
**MIP-1δ**	**0.42**	0.83	0.52	0.70	1.30	0.8822	0.1576
**RANTES**	1.12	1.09	**1.60**	**1.71**	1.32	0.0200	0.0142
**SCF**	0.53	0.62	0.58	0.94	1.38	0.8807	0.2023
**SDF-1**	0.88	0.90	0.85	1.17	1.19	0.8917	0.9051
**TARC**	**2.23**	2.04	**1.71**	1.33	2.09	0.4584	0.4890
**TGF-β1**	1.17	1.15	1.17	1.21	1.44	0.9385	0.9937
**TNF-α**	**12.52**	**9.47**	**15.51**	**14.74**	**9.47**	0.0193	0.0412
**TNF-β**	0.96	1.00	1.10	1.07	0.72	0.8319	0.8839
**EGF**	0.90	1.01	0.74	0.74	0.95	0.8789	0.7227
**IGF-I**	0.76	0.87	0.83	0.84	1.12	0.9997	0.4810
**Angiogenin**	5.99	**6.22**	2.75	1.65	1.29	0.2959	0.4104
**Oncostatin M**	0.79	1.05	0.77	0.97	1.46	0.8613	0.8078
**Thrombopoietin**	**2.08**	2.13	**2.06**	**2.07**	1.35	0.3792	0.4682
**VEGF**	0.88	0.87	0.75	0.93	1.44	0.8574	0.6237
**PDGF-BB**	1.35	1.19	0.80	1.22	**1.94**	0.0095	0.0568
**Leptin**	0.88	0.73	1.07	1.56	1.59	0.7187	0.8510

	GB112	GB411	GB590	GB653	GB37	p-value densitometry	p-value fold change
BNDF	0.91	0.89	0.94	0.91	0.89	0.9958	0.9088
BLC	**5.50**	**6.70**	**4.51**	**2.32**	**2.79**	0.5415	0.4038
Ck β 8-1	1.52	**2.01**	1.29	1.81	1.41	0.3255	0.3678
Eotaxin	1.47	1.89	**1.60**	1.84	1.37	0.5811	0.5195
Eotaxin-2	1.78	**1.64**	2.07	**2.19**	1.57	0.4096	0.6670
Eotaxin-3	1.53	1.85	1.03	**2.50**	1.22	0.2288	0.2142
FGF-4	0.96	1.03	1.62	1.81	1.31	0.3589	0.9119
FGF-6	1.50	1.45	1.25	1.92	1.54	0.8200	0.9820
FGF-7	1.34	1.43	1.09	1.70	1.38	0.4690	0.9703
FGF-9	1.36	1.33	1.17	1.74	1.07	0.9014	0.4406
Flt-3 Ligand	**1.87**	**2.79**	**2.09**	**2.52**	1.94	0.1610	0.1134
Fractalkine	1.29	**1.91**	1.14	1.49	1.19	0.5702	0.1606
GCP-2	1.73	**2.29**	1.39	1.60	1.42	0.6218	0.2889
GDNF	1.01	1.73	1.12	1.49	1.27	0.5716	0.4613
HGF	**2.10**	**3.33**	2.30	**3.20**	**2.01**	0.2369	0.2853
IGFBP-1	1.15	**1.99**	0.88	2.52	1.26	0.2619	0.5099
IGFBP-2	1.14	1.38	1.15	1.53	0.88	0.9103	0.4336
IGFBP-3	1.07	0.94	1.45	1.50	1.11	0.7520	0.9580
IGFBP-4	2.03	1.69	1.65	2.07	1.02	0.7380	0.8207
IL-16	0.66	1.01	1.00	1.12	0.87	0.8858	0.9831
IP-10	**6.73**	**5.20**	**5.24**	**6.41**	**2.43**	0.6158	0.0001
LIF	0.84	0.68	0.73	0.90	1.19	0.9741	0.6426
LIGHT	0.91	1.11	1.22	1.22	1.15	0.5146	0.6999
MCP-4	1.62	2.49	2.62	2.95	**2.32**	0.1698	0.8278
MIF	**2.17**	**2.09**	**1.91**	**2.89**	**2.00**	0.7370	0.1826
MIP-3α	**6.30**	**4.77**	**6.40**	**6.44**	**2.69**	0.7039	0.2847
NAP-2	1.28	1.70	1.60	1.93	**2.13**	0.1869	0.8593
NT-3	1.33	**2.45**	2.41	**1.76**	1.30	0.9783	0.2990
NT-4	0.68	**0.64**	1.43	1.23	1.05	0.5148	0.8519
Osteopontin	0.69	0.69	1.75	1.48	0.75	0.7771	0.1847
Osteoprotegerin	0.85	0.92	0.74	0.89	1.23	0.6404	0.1220
PARC	**4.57**	**4.58**	1.84	2.45	2.67	0.5280	0.3646
PLGF	2.73	**2.47**	1.15	1.51	1.92	0.5363	0.6843
TGF-β2	1.05	0.88	1.17	1.41	0.76	0.9708	0.0605
TGF-β3	1.84	**1.69**	1.14	1.20	1.21	0.7090	0.6990
TIMP-1	0.87	0.88	1.01	0.98	1.02	0.9999	0.6279
TIMP-2	0.84	3.34	1.11	1.20	**0.48**	0.8293	0.5811

Heat Map Key 0.20 1.00 15.00
