# Supplementary figures and images for: Genetically distinct Group B Streptococcus strains induce varying macrophage cytokine responses
Source: PLoS One. 2019 Sep 19;14(9):e0222910. doi: 10.1371/journal.pone.0222910 (PMC6752832; doi:10.1371/journal.pone.0222910)

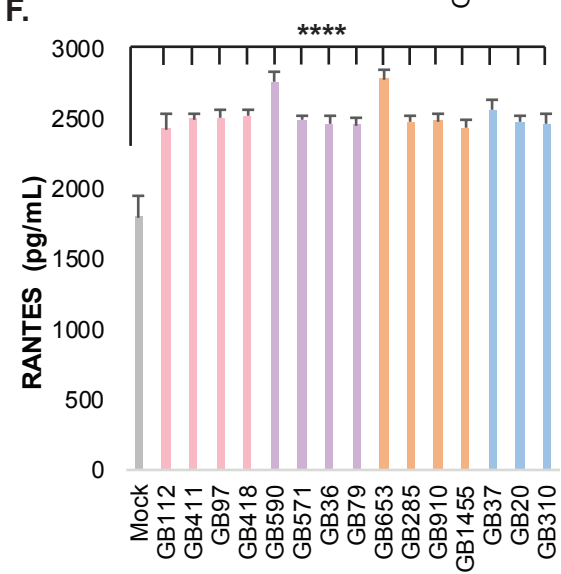

Supplement: S4 Fig — THP-1 cells were infected with GBS at MOI of 10 for 1hr. Data from three to five independent biological replicates were pooled to determine the average cytokine concentrations (pg/mL) produced following each of the infection conditions shown. The average and standard deviation of each condition were plotted for comparison, and significance was determined by ANOVA, followed by post-ANOVA Dunnett’s tests to compare the mean of each condition to the mean of the mock infection condition. All cytokines tested had ANOVA p-values <0.0001. (PDF) [file pone.0222910.s004.pdf]

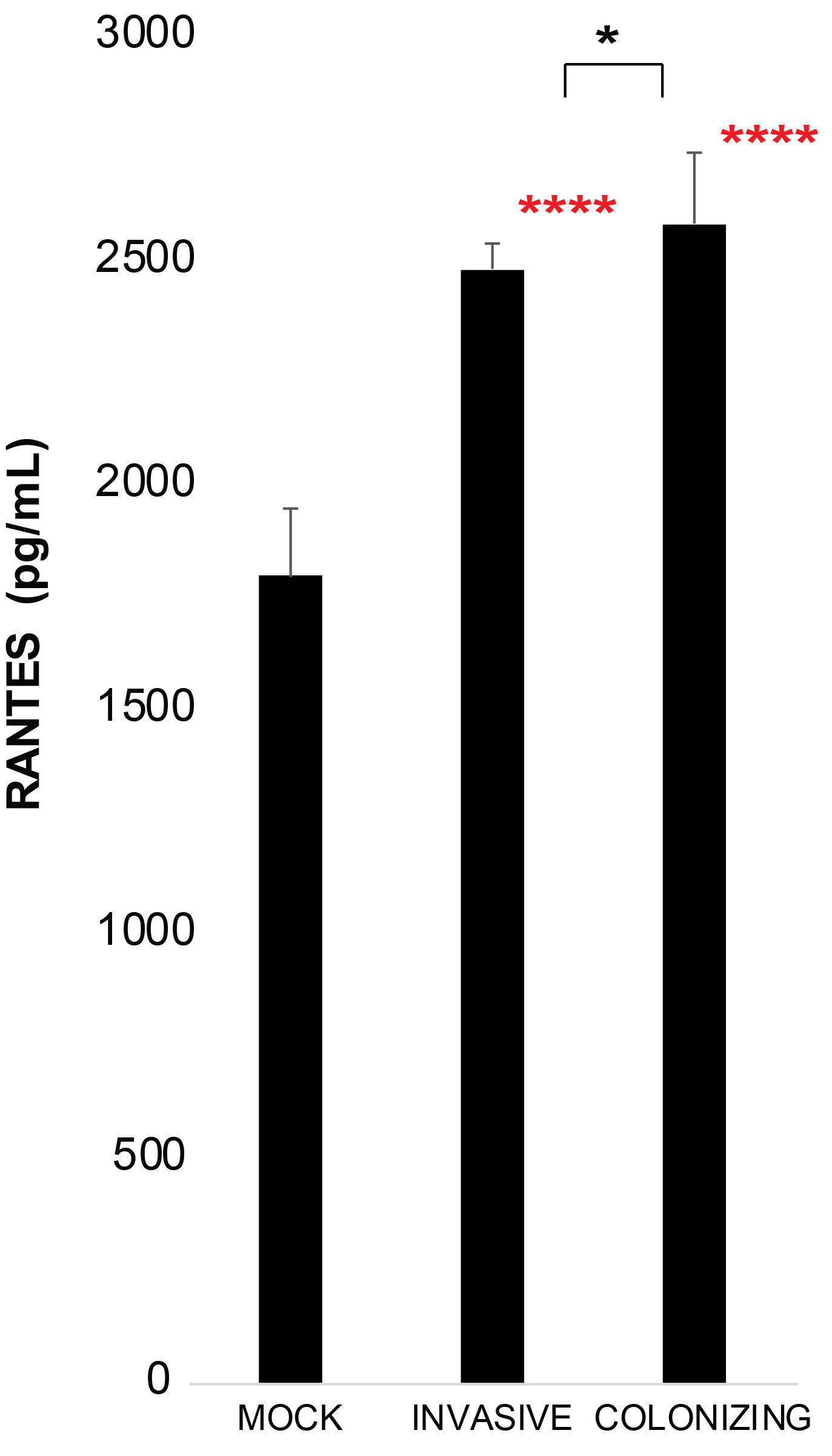

Supplement: S5 Fig — THP-1 cells were infected with GBS at MOI of 10 for 1hr. Data from three to five independent biological replicates were pooled to calculate the average RANTES concentration (pg/mL) produced following infection with invasive or colonizing GBS strains. The standard deviation was plotted for comparison, and significance was determined by ANOVA using Tukey’s test of multiple comparisons. p-values <0.0001 are indicated. (JPG) [file pone.0222910.s005.jpg]
