# Supplementary material for: Genetically distinct Group B Streptococcus strains induce varying macrophage cytokine responses
Source: PLoS One. 2019 Sep 19;14(9):e0222910. doi: 10.1371/journal.pone.0222910 (PMC6752832; doi:10.1371/journal.pone.0222910)
Supplement: S6 Fig — Cytokines with increased production relative to mock infection in both cytokine array replicates during infection with GBS strains are shown in red, and cytokines with decreased production are shown in blue. Values represent the average fold change relative to mock infection from the two independent array replicates for each condition. Bolded values represent cytokines that had fold changes of ≥1.5 above or below the mock infection in both array replicates. Major cytokine functions were classified via the UniProt Database (http://www.uniprot.org/). (PDF) [file pone.0222910.s006.pdf]

Granulocyte Activators & Chemokines

	GB112	GB411	GB590	GB653	GB37
**ENA-78**	1.54	**2.16**	1.58	1.39	1.62
**GRO**	**1.95**	1.59	1.88	1.84	**1.60**
**GRO-α**	**3.33**	**3.36**	**5.73**	**5.02**	**3.59**
**IL-3**	1.70	1.60	**2.00**	**1.68**	1.44
**MCP-2**	**15.82**	**14.68**	**9.99**	**6.66**	**5.06**
**MIP-1δ**	**0.42**	0.83	0.52	0.70	1.30
**Ck β 8-1**	1.52	**2.01**	1.29	1.81	1.41
**Eotaxin**	1.47	1.89	**1.60**	1.84	1.37
**Eotaxin-2**	1.78	**1.64**	2.07	**2.19**	1.57
**Eotaxin-3**	1.53	1.85	1.03	**2.50**	1.22
**GCP-2**	1.73	**2.29**	1.39	1.60	1.42
**MCP-4**	1.62	2.49	2.62	2.95	**2.32**
**MIP-3α**	**6.30**	**4.77**	**6.40**	**6.44**	**2.69**
**NAP-2**	1.28	1.70	1.60	1.93	**2.13**

Monocyte and Dendritic Cell Activators & Chemokines

	GB112	GB411	GB590	GB653	GB37
**IL-3**	1.70	1.60	**2.00**	**1.68**	1.44
**MCP-1**	**2.00**	1.64	**2.84**	**2.48**	**1.85**
**MCP-2**	**15.82**	**14.68**	**9.99**	**6.66**	**5.06**
**MIP-1δ**	**0.42**	0.83	0.52	0.70	1.30
**RANTES**	1.12	1.09	**1.60**	**1.71**	1.32
**Ck β 8-1**	1.52	**2.01**	1.29	1.81	1.41
**Flt-3 Ligand**	**1.87**	**2.79**	**2.09**	**2.52**	1.94
**Fractalkine**	1.29	**1.91**	1.14	1.49	1.19
**IP-10**	**6.73**	**5.20**	**5.24**	**6.41**	**2.43**
**MIF**	**2.17**	**2.09**	**1.91**	**2.89**	**2.00**
**MCP-4**	1.62	2.49	2.62	2.95	**2.32**
**MIP-3α**	**6.30**	**4.77**	**6.40**	**6.44**	**2.69**

Regulators of Growth, Differentiation, and Survival

	GB112	GB411	GB590	GB653	GB37
**Thrombopoietin**	**2.08**	2.13	**2.06**	**2.07**	1.35
**PDGF-BB**	1.35	1.19	0.80	1.22	**1.94**
**HGF**	**2.10**	**3.33**	2.30	**3.20**	**2.01**
**IGFBP-1**	1.15	**1.99**	0.88	2.52	1.26
**NT-3**	1.33	**2.45**	2.41	**1.76**	1.30
**PLGF**	2.73	**2.47**	1.15	1.51	1.92
**TGF-β3**	1.84	**1.69**	1.14	1.20	1.21

Metalloproteinase inhibitors

	GB112	GB411	GB590	GB653	GB37
**TIMP-2**	0.84	3.34	1.11	1.20	**0.48**

Regulators of Vascularization of Tissues

	GB112	GB411	GB590	GB653	GB37
**Angiogenin**	5.99	**6.22**	2.75	1.65	1.29
**IP-10**	**6.73**	**5.20**	**5.24**	**6.41**	**2.43**
**PLGF**	2.73	**2.47**	1.15	1.51	1.92

Multifunctional Pro-inflammatory Cytokines

	GB112	GB411	GB590	GB653	GB37
**I-309**	**4.05**	**3.62**	**2.91**	**2.81**	**3.32**
**IL-1β**	**2.32**	**2.35**	**3.36**	**3.13**	**2.51**
**IL-6**	**6.14**	**4.91**	**4.85**	**4.08**	**3.70**
**TNF-α**	**12.52**	**9.47**	**15.51**	**14.74**	**9.47**
**MIF**	**2.17**	**2.09**	**1.91**	**2.89**	**2.00**

Multifunctional Anti-inflammatory Cytokines

	GB112	GB411	GB590	GB653	GB37
**IL-10**	5.64	**14.94**	2.74	2.08	1.52
**IL-13**	1.14	**2.21**	1.59	1.40	1.26

B-Cell Activators & Chemokines

	GB112	GB411	GB590	GB653	GB37
**BLC**	**5.50**	**6.70**	**4.51**	**2.32**	**2.79**
**MCP-4**	1.62	2.49	2.62	2.95	**2.32**
**MIP-3α**	**6.30**	**4.77**	**6.40**	**6.44**	**2.69**
**PARC**	**4.57**	**4.58**	1.84	2.45	2.67

T-Cell and NK Cell Activators & Chemokines

	GB112	GB411	GB590	GB653	GB37
**IL-12**	0.70	0.84	0.84	0.75	**2.22**
**MCP-1**	**2.00**	1.64	**2.84**	**2.48**	**1.85**
**MCP-2**	**15.82**	**14.68**	**9.99**	**6.66**	**5.06**
**MIG**	**11.63**	**7.39**	5.26	2.13	1.52
**MIP-1δ**	**0.42**	0.83	0.52	0.70	1.30
**RANTES**	1.12	1.09	**1.60**	**1.71**	1.32
**TARC**	**2.23**	2.04	**1.71**	1.33	2.09
**Ck β 8-1**	1.52	**2.01**	1.29	1.81	1.41
**Fractalkine**	1.29	**1.91**	1.14	1.49	1.19
**IP-10**	**6.73**	**5.20**	**5.24**	**6.41**	**2.43**
**MCP-4**	1.62	2.49	2.62	2.95	**2.32**
**MIP-3α**	**6.30**	**4.77**	**6.40**	**6.44**	**2.69**
**PARC**	**4.57**	**4.58**	1.84	2.45	2.67

Regulators of Embryonic and Placental Development

	GB112	GB411	GB590	GB653	GB37
**Flt-3 Ligand**	**1.87**	**2.79**	**2.09**	**2.52**	1.94
**PLGF**	2.73	**2.47**	1.15	1.51	1.92
**TGF-β3**	1.84	**1.69**	1.14	1.20	1.21

Regulators of Platelet Production

	GB112	GB411	GB590	GB653	GB37
**Thrombopoietin**	**2.08**	2.13	**2.06**	**2.07**	1.35

Regulators of Nervous System Cells

	GB112	GB411	GB590	GB653	GB37
**NT-3**	1.33	**2.45**	2.41	**1.76**	1.30

Hematopoietic Precursor Activators

	GB112	GB411	GB590	GB653	GB37
**GM-CSF**	**2.59**	**2.94**	**2.60**	**2.30**	1.54

Heat Map Key: 0.20 | 1.00 | 15.00
