# Supplementary material for: Genetically distinct Group B Streptococcus strains induce varying macrophage cytokine responses
Source: PLoS One. 2019 Sep 19;14(9):e0222910. doi: 10.1371/journal.pone.0222910 (PMC6752832; doi:10.1371/journal.pone.0222910)
Supplement: S1 Table — Major functions were defined by and extracted from the UniProt Database (http://www.uniprot.org/). Abbreviations: NKs–Natural Killer Cells; DCs–Dendritic Cells; MMP–Matrix Metalloproteinases; TIMPs–Tissue inhibitors of metalloproteinases; SC–Stem Cells. (PDF) [file pone.0222910.s007.pdf]

Table S1: Functional annotations of cytokines examined in the study. Major functions were defined by and extracted from the UniProt Database (http://www.uniprot.org/). **Abbreviations:** NKs – Natural Killer Cells; DCs – Dendritic Cells; MMP – Matrix Metalloproteinases; TIMPs – Tissue inhibitors of metalloproteinases; SC – Stem Cells.

Cytokine	Complete cytokine name	Major Cytokine Functions
**ENA-78**	Epithelial neutrophil activating peptide-78 (CXCL5)	Activates neutrophils.
**GCSF**	Granulocyte colony stimulating factor	Stimulates the production, differentiation, and function of granulocytes.
**GM-CSF**	Granulocyte-monocyte colony stimulating factor	Stimulates the growth and differentiation of hematopoietic precursors.
**GRO**	Growth regulated oncogene	Chemotactic for neutrophils.
**GRO-α**	Growth regulated oncogene alpha	Chemotactic for neutrophils.
**I-309**	CC motif chemokine ligand 1 (CCL1)	Potent pro-inflammatory cytokine; activates numerous immune cell types, particularly monocytes.
**IL-1α**	Interleukin-1 alpha	Multifunctional and potent pro-inflammatory cytokine.
**IL-1β**	Interleukin-1 beta	Potent pro-inflammatory cytokine; activates numerous immune cell types.
**IL-2**	Interleukin-2	Aids in tolerance and immunity; promotes the production of regulatory T-Cells.
**IL-3**	Interleukin-3	Controls production, differentiation, and function of granulocytes, monocytes and macrophages.
**IL-4**	Interleukin-4	Activates B-Cells.
**IL-5**	Interleukin-5	Stimulates B-Cell growth, antibody production, and eosinophil activation.
**IL-6**	Interleukin-6	Multifunctional and potent pro-inflammatory cytokine.
**IL-7**	Interleukin-7	Hematopoietic growth factor; stimulates proliferation of lymphoid progenitors.
**IL-8**	Interleukin-8	Chemotactic for neutrophils.
**IL-10**	Interleukin-10	Inhibitor of several pro-inflammatory cytokines.
**IL-12**	Interleukin-12	Growth factor for activated T-Cells and NK cells.
**IL-13**	Interleukin-13	Inhibits inflammatory cytokine production.
**IL-15**	Interleukin-15	Activates T-Cells and NK cells.
**IFN-γ**	Interferon gamma	Activates macrophages.
**MCP-1**	Monocyte chemoattractant protein 1 (CCL2)	Recruits monocytes, memory T-Cells, and DCs to sites of inflammation.
**MCP-2**	Monocyte chemoattractant protein 2 (CCL8)	Chemotactic factor for monocytes, lymphocytes, basophils and eosinophils.
**MCP-3**	Monocyte chemoattractant protein 3 (CCL7)	Chemotactic factor that attracts monocytes and eosinophils.

**MCSF**	Macrophage colony stimulating factor (CSF1)	Regulates survival, proliferation and differentiation of hematopoietic precursors; promotes pro-inflammatory cytokine release.
**MDC**	CC motif chemokine ligand 22 (CCL22)	Regulates trafficking of activated effector T-Cells to inflammatory sites; chemotactic for monocytes, DCs and NKs.
**MIG**	Monokine induced by gamma interferon (CXCL9)	Chemotactic for T-Cells.
**MIP-1b**	Macrophage inflammatory protein 1 beta (CCL4)	Monokine with inflammatory and chemokinetic properties.
**MIP-1δ**	Macrophage inflammatory protein 1 delta (CCL15)	Chemotactic factor that attracts T-Cells and monocytes.
**RANTES**	Regulated on activation, normal T cell expressed and secreted (CCL5)	Chemoattractant for blood monocytes, memory T-helper cells and eosinophils.
**SCF**	Stem cell factor (kit ligand)	Regulates survival, proliferation, hematopoiesis, SC maintenance, gametogenesis, mast cell development, migration and function.
**SDF-1**	Stromal cell derived factor 1 (CXCL12)	Chemoattractant for T-lymphocytes and monocytes.
**TARC**	T cell directed CC chemokine (CCL17)	Chemotactic factor for T-lymphocytes.
**TGF-β1**	Transforming growth factor beta 1	Controls proliferation, differentiation and other functions in many cell types.
**TNF-α**	Tumor necrosis factor alpha	Potent pyrogen; mainly secreted by macrophages and can induce cell death.
**TNF-β**	Tumor necrosis factor beta	Involved in the regulation of cell survival, proliferation, differentiation, and apoptosis.
**EGF**	Epidermal growth factor	Stimulates the growth of various epidermal and epithelial tissues in vivo and in vitro and of some fibroblasts in cell culture.
**IGF-I**	Insulin like growth factor 1	Promotes growth via activation of PI3K-AKT/PKB and Ras-MAPK pathways.
**Angiogenin**	Angiogenin	Induces vascularization of tissues.
**Oncostatin M**	Oncostatin M	Growth regulator. Inhibits tumor cell proliferation and regulates cytokine production, including IL-6, G-CSF and GM-CSF.
**Thrombopoietin**	Thrombopoietin	Regulates the production of platelets by stimulating megakaryocytes, the bone marrow cells that produce platelets.
**VEGF**	Vascular endothelial growth factor	Growth factor active in angiogenesis, vasculogenesis and endothelial cell growth.
**PDGF-BB**	Platelet derived growth factor (B subunits)	Potent mitogen for cells of mesenchymal origin, including fibroblasts, smooth muscle cells and glial cells.
**Leptin**	Leptin	Pro-inflammatory, pro-angiogenic, mitogenic, regulates MMPs and TIMPs, recruits/activates neutrophils, macrophages, NKs and T-Cells.
**BNDF**	Brain derived neurotrophic factor	Supports the survival of neurons, and encourages growth and differentiation of new neurons and synapses.
**BLC**	B cell lymphoma 2	Selectively chemotactic for B cells belonging to both the B-1 and B-2 subsets.
**Ck β 8-1**	CC motif chemokine ligand 23 (CCL23)	Chemotactic for resting T-lymphocytes and monocytes, and has some effect on neutrophils.
**Eotaxin**	Eotaxin	Chemotactic for eosinophils.
**Eotaxin-2**	Eotaxin-2	Chemotactic for eosinophils.
**Eotaxin-3**	Eotaxin-3	Chemotactic for eosinophils and basophils.
**FGF-4**	Fibroblast growth factor-4	Regulates embryonic development, cell proliferation, and differentiation.
**FGF-6**	Fibroblast growth factor-6	Chemoattractant for eosinophils and basophils.
**FGF-7**	Fibroblast growth factor-7	Member of the fibroblast growth factor (FGF) family and has broad mitogenic and cell survival activities.
**FGF-9**	Fibroblast growth factor-9	Regulates embryonic development, cellular proliferation, differentiation, migration and repair of some cell types including glial and neuronal cells.
**Flt-3 Ligand**	FMS-like tyrosine kinase 3 ligand	Activates macrophages and involved in placental development.
**Fractalkine**	Fractalkine	Chemotactic for T-cells and monocytes.

**GCP-2**	Granulocyte chemotactic protein 2	Chemoattractant for neutrophils.
**GDNF**	Glial cell derived neurotrophic factor	Potently promotes the survival of many types of neurons.
**HGF**	Hepatocyte growth factor	Potent mitogen for hepatocytes, hepatotrophic, growth factor for many tissues and cell types, and is the ligand for MET.
**IGFBP-1**	Insulin-like growth factor binding protein 1	Prolongs the half-life of the IGFs; can stimulate or inhibit cell growth.
**IGFBP-2**	Insulin-like growth factor binding protein 2	Inhibits IGF-mediated growth and developmental rates. Prolongs the half-life of the IGFs and can either inhibit or stimulate growth.
**IGFBP-3**	Insulin-like growth factor binding protein 3	Prolongs the half-life of the IGFs; can stimulate or inhibit cell growth.
**IGFBP-4**	Insulin-like growth factor binding protein 4	Prolongs the half-life of the IGFs; can stimulate or inhibit cell growth.
**IL-16**	Interleukin 16	Attracts activated T-Cells; recruits and activates many other cells expressing CD4, including monocytes, eosinophils, and dendritic cells.
**IP-10**	Interferon gamma induced protein 10 (CXCL10)	Chemotactic for monocytes/macrophages, T-Cells, NK cells, and DCs; inhibits bone marrow colony formation and angiogenesis.
**LIF**	Leukemia inhibitory factor	Induces cell differentiation in hematopoietic cells, myeloid cells, and neuronal cells; stimulates acute-phase protein synthesis in hepatocytes.
**LIGHT**	Tumor necrosis factor superfamily member 14 (TNFSF14)	Costimulatory factor for the activation of lymphoid cells, particularly T-Cells.
**MCP-4**	Monocyte chemotactic protein 4 (CCL13)	Chemotactic factor for monocytes, lymphocytes, basophils and eosinophils.
**MIF**	Macrophage migration inhibitory factor	Pro-inflammatory cytokine. Involved in the innate immune response to bacterial pathogens likely through regulation of macrophage function.
**MIP-3α**	Macrophage inflammatory protein 3 alpha	Chemotactic for DCs, neutrophils, T-Cells and B-Cells.
**NAP-2**	Neutrophil activating peptide 2	Ligand for CXCR1 and CXCR2; chemoattractant and activator for neutrophils.
**NT-3**	Neurotrophin-3	Growth factor with activity on certain neurons of the peripheral and central nervous system.
**NT-4**	Neurotrophin-4	Survival factor for peripheral sensory sympathetic neurons.
**Osteopontin**	Osteopontin	Adhesion protein; chemotactic and stimulatory for multiple immune cell types; also regulates attachment, wound healing, and apoptosis.
**Osteoprotegerin**	Osteoprotegerin	Inhibits the activation of osteoclasts and promotes osteoclast apoptosis in vitro.
**PARC**	Pulmonary and activation-regulated chemokine (CCL18)	Chemotactic factor for T and B lymphocytes; guides migration to lymph nodes. Attracts naive T-Cells to DCs and activated macrophages.
**PLGF**	Placental growth factor	Member of the VEGF family; has roles in development, tissue ischemia, malignancy, inflammation, and multiple other diseases.
**TGF-β2**	Transforming growth factor beta 2	Has suppressive effects on IL-2 dependent T-Cell growth. Performs many cellular functions and is critical for embryonic development.
**TGF-β3**	Transforming growth factor beta 3	Involved in embryogenesis and cell differentiation.
**TIMP-1**	Tissue inhibitor of metalloproteinases 1	Metalloproteinase inhibitor that acts on MMP 1-3, MMP7-13, and MMP16. Also regulates cell differentiation, migration and cell death and signaling.
**TIMP-2**	Tissue inhibitor of metalloproteinases 2	Metalloproteinase inhibitor that acts on MMP 1-3, MMP 7-10, MMP 13-16 and MMP-19.
