# Supplementary material for: Genetically distinct Group B Streptococcus strains induce varying macrophage cytokine responses
Source: PLoS One. 2019 Sep 19;14(9):e0222910. doi: 10.1371/journal.pone.0222910 (PMC6752832; doi:10.1371/journal.pone.0222910)
Supplement: S2 Fig — Differentiated THP-1 cells were infected with GBS strains for 1hr at a MOI of 10 bacteria per host cell. Densitometry values for each cytokine are shown from each of the two array replicates. The internal positive control values were averaged for individual arrays and used to normalize each array condition to allow accurate comparisons between mock and infection conditions. Negative control values from each array are also shown to indicate the lower limit of detection for the array. (PDF) [file pone.0222910.s002.pdf]

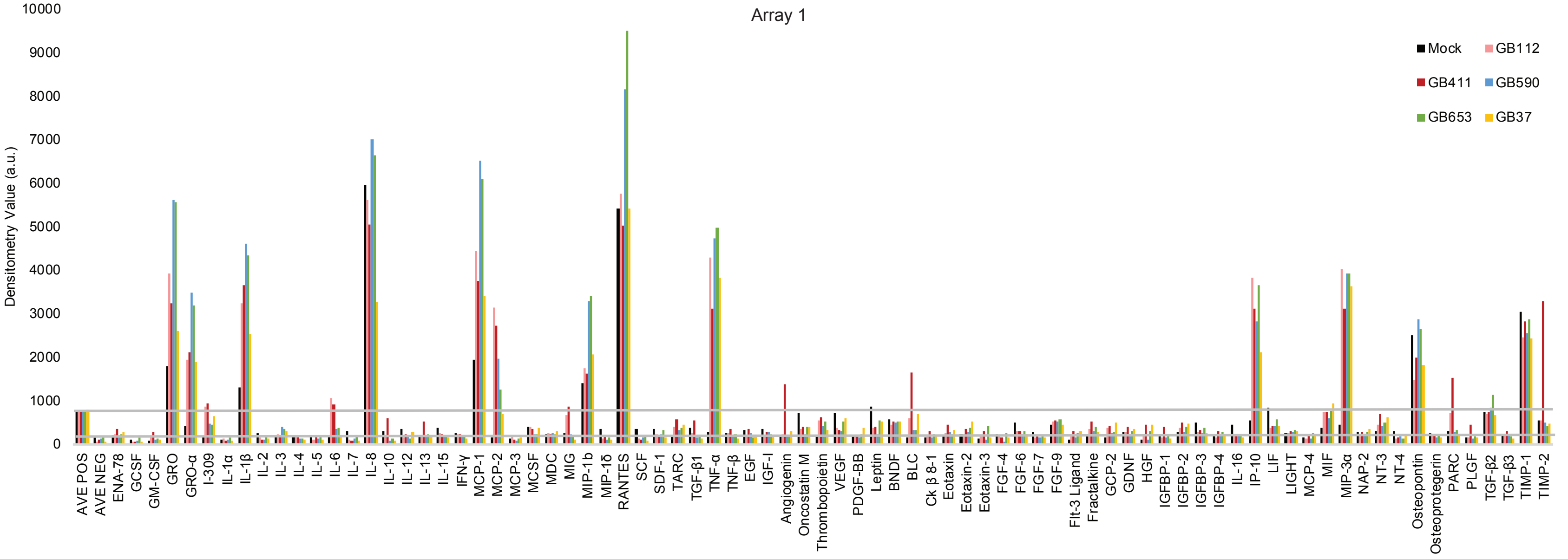
Array 1
Densitometry Value (a.u.)
0
1000
2000
3000
4000
5000
6000
7000
8000
9000
10000
Mock
GB112
GB411
GB590
GB653
GB37
AVE POS
AVE NEG
ENA-78
GCSF
GM-CSF
GRO
GRO-α
I-309
IL-1α
IL-1β
IL-2
IL-3
IL-4
IL-5
IL-6
IL-7
IL-8
IL-10
IL-12
IL-13
IL-15
IFN-γ
MCP-1
MCP-2
MCP-3
MCSF
MDC
MIG
MIP-1b
MIP-1δ
RANTES
SCF
SDF-1
TARC
TGF-β1
TNF-α
TNF-β
EGF
IGF-I
Angiogenin
Oncostatin M
Thrombopoietin
VEGF
PDGF-BB
Leptin
BNDF
BLC
Ck β 8-1
Eotaxin
Eotaxin-2
Eotaxin-3
FGF-4
FGF-6
FGF-7
FGF-9
Flt-3 Ligand
Fractalkine
GCP-2
GDNF
HGF
IGFBP-1
IGFBP-2
IGFBP-3
IGFBP-4
IL-16
IP-10
LIF
LIGHT
MCP-4
MIF
MIP-3α
NAP-2
NT-3
NT-4
Osteopontin
Osteoprotegerin
PARC
PLGF
TGF-β2
TGF-β3
TIMP-1
TIMP-2

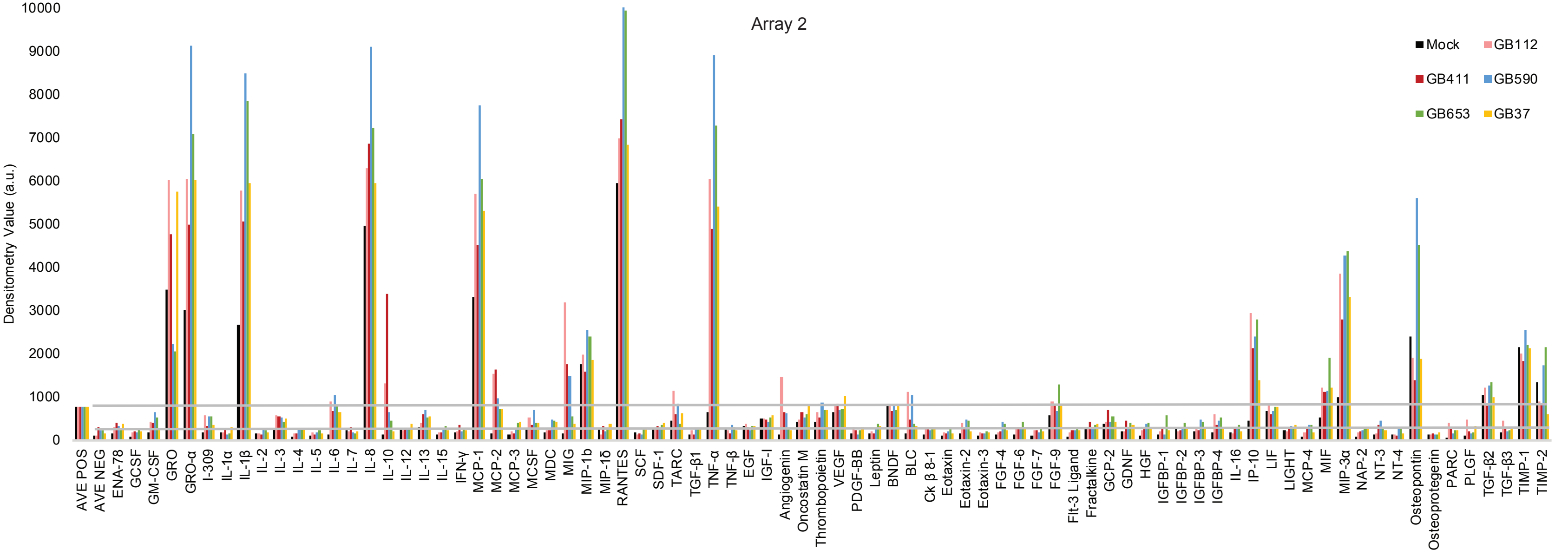
Array 2
Densitometry Value (a.u.)
0
1000
2000
3000
4000
5000
6000
7000
8000
9000
10000
Mock
GB112
GB411
GB590
GB653
GB37
AVE POS
AVE NEG
ENA-78
GCSF
GM-CSF
GRO
GRO-α
I-309
IL-1α
IL-1β
IL-2
IL-3
IL-4
IL-5
IL-6
IL-7
IL-8
IL-10
IL-12
IL-13
IL-15
IFN-γ
MCP-1
MCP-2
MCP-3
MCSF
MDC
MIG
MIP-1b
MIP-1δ
RANTES
SCF
SDF-1
TARC
TGF-β1
TNF-α
TNF-β
EGF
IGF-I
Angiogenin
Oncostatin M
Thrombopoietin
VEGF
PDGF-BB
Leptin
BNDF
BLC
Ck β 8-1
Eotaxin
Eotaxin-2
Eotaxin-3
FGF-4
FGF-6
FGF-7
FGF-9
Flt-3 Ligand
Fractalkine
GCP-2
GDNF
HGF
IGFBP-1
IGFBP-2
IGFBP-3
IGFBP-4
IL-16
IP-10
LIF
LIGHT
MCP-4
MIF
MIP-3α
NAP-2
NT-3
NT-4
Osteopontin
Osteoprotegerin
PARC
PLGF
TGF-β2
TGF-β3
TIMP-1
TIMP-2
